# Aberrations and adaptive optics in super-resolution microscopy

**DOI:** 10.1093/jmicro/dfv033

**Published:** 2015-06-28

**Authors:** Martin Booth, Débora Andrade, Daniel Burke, Brian Patton, Mantas Zurauskas

**Affiliations:** 1 Centre for Neural Circuits and Behaviour, University of Oxford, Tinsley Building, Mansfield Road, Oxford OX1 3SR, UK; 2 Department of Engineering Science, University of Oxford, Parks Road, Oxford OX1 3PJ, UK

**Keywords:** adaptive optics, aberrations, super-resolution microscopy, single-molecule switching, stimulated emission depletion, structured illumination

## Abstract

As one of the most powerful tools in the biological investigation of cellular structures and dynamic processes, fluorescence microscopy has undergone extraordinary developments in the past decades. The advent of super-resolution techniques has enabled fluorescence microscopy – or rather nanoscopy – to achieve nanoscale resolution in living specimens and unravelled the interior of cells with unprecedented detail. The methods employed in this expanding field of microscopy, however, are especially prone to the detrimental effects of optical aberrations. In this review, we discuss how super-resolution microscopy techniques based upon single-molecule switching, stimulated emission depletion and structured illumination each suffer from aberrations in different ways that are dependent upon intrinsic technical aspects. We discuss the use of adaptive optics as an effective means to overcome this problem.

## Introduction

All super-resolution microscopes are susceptible to the effects of aberrations, especially when focussing deep into specimens. The aberrations arise when light propagates through regions of the specimen that have different refractive index. Common sources include the mismatch in refractive index between the mounting medium and the cover glass or the variations in refractive index that exist throughout the different components of the cell or throughout the broader structure of tissue specimens. Aberrations have different effects in each type of microscope, but in all cases lead to reduced image contrast and loss of resolution. In extreme situations, the consequence could be such a large reduction in efficiency that a useful image is essentially unachievable.

Adaptive optics (AO) has been introduced into a range of microscopes to correct aberrations and restore image quality [1–3]. The principle behind an AO system is the measurement and correction of aberrations, usually in closed loop, by combination of an adaptive correction element, such as a deformable mirror (DM) or spatial light modulator (SLM), and a method of wavefront measurement. AO has been implemented in various types of conventional microscopes, but recent developments have brought AO into use for far-field super-resolution optical microscopes.

Super-resolution microscopes exist in different manifestations, but they all rely upon the highest quality optical systems, so are generally much more sensitive to aberration effects. This means that AO has a major role to play in making the use of super-resolution more widespread, particularly when used for imaging deep in specimens. However, it also means that there are significant challenges in the implementation.

In this review, we discuss the specific ways in which aberrations affect these super-resolution microscopes and the considerations that should be made in implementation of AO in these systems.

## Super-resolution microscopy

We consider three types of fluorescence-based super-resolution microscopy: single-molecule switching (SMS) microscopy [4–6], stimulated emission depletion (STED) microscopy [7] and structured illumination microscopy (SIM) [8]. These complementary methods have facilitated a revolution in the way that light microscopes can be used to probe biological processes. As there is extensive discussion of these methods in the cited literature, we provide here just a brief summary of the operating principles.

SMS microscopy is an umbrella term that encompasses methods such as stochastic optical reconstruction microscopy (STORM), photoactivation localization microscopy (PALM), ground-state depletion microscopy (GSDIM) and many other variants. In all of these microscopes, the image is formed following similar principles. During imaging, the fluorescent markers are caused to switch on and off in such a way that only a small proportion are emitting in any one image frame. On average, each emitter is well separated from the others, so it is possible to locate the position of the emitter to a precision far smaller than the size of the microscope's point spread function (PSF). Through acquisition of many thousands of image frames, one accumulates the positions of large numbers of emitters. From these data, an image representation with localization precision in the order of 10 nm can be constructed.

STED microscopy is a laser scanning method, in which super-resolution is achieved by confining the fluorescence emission to a region much smaller than the focussed laser beam. Fluorescent molecules are excited, as in a confocal microscope, using a suitable laser focussed to a diffraction-limited spot by a high numerical aperture objective lens. Superimposed upon this is the focus of a second laser, which is shaped to have a zero intensity point at the centre of a bright ring of illumination. This beam forces the excited fluorophores to undergo stimulated emission, thus preventing them from emitting detectable fluorescence, except at the centre of the depletion beam, where the intensity was zero. As the stimulated emission process can be saturated, it is possible to reduce the size of the region of remaining fluorescence by increasing the intensity of the depletion beam. The resolution of the resulting image is much smaller than the size of the original excitation focus, reportedly around 20 nm (*xy* plane) in biological specimens [9,10].

SIM extends the capabilities of widefield fluorescence microscopes by using patterned illumination to access higher spatial frequencies that are not normally seen by a standard microscope. Only spatial frequencies up to a maximum cut-off can be imaged by a widefield microscope. However, if illuminated by a structure consisting of a sinusoidal pattern of bright and dark stripes at the cut-off frequency, higher frequencies up to twice the cut-off can be encoded in the image. Through acquisition of a sequence of images with different orientations and positions of the structured illumination pattern, and followed by appropriate processing, it is possible to extend the range of spatial frequencies by a factor of two in all directions.

## Adaptive optics correction methods

The two main devices for aberration correction are DMs and SLMs. DMs consist of a flexible reflective surface that can be distorted through the application of forces from an array of actuators. These DMs are usually membrane or microstructured mirrors with continuous mirror surfaces that are electrostatically or electromagnetically actuated. Segmented DMs have also been used. A DM controls wavefront phase by changing the relative optical path length (OPL) between different parts of the beam as it is reflected. DMs are polarization insensitive, and their wavelength dependence is similar to that of normal mirrors.

It should be noted that a DM introduces an aberration into the optical system by modifying the OPL by a distance equal to twice the mirror surface deformation. Consequently, the phase aberration introduced by the DM varies in the inverse proportion to wavelength. It may seem, therefore, that the DM provides chromatically dependent correction. However, one should also consider the source of the aberrations in the system. Aberrations induced by the specimen are caused by its refractive index structure, and this structure modifies the OPL. It follows that the phase aberrations from the specimen also scale inversely with wavelength, so the compensation provided by the DM varies in exactly the same way. Discrepancies could arise if the specimen is dispersive, but this is not common in biological specimens.

SLMs are usually pixelated liquid crystal devices that change the OPL by modification of the effective refractive index of each pixel. These devices tend to have a much larger number of pixels than the number of actuators available on a DM. For that reason, SLMs are able to create more complex phase distributions. However, the pixels are usually configured to provide 2*π* radians of phase modulation. This means that larger amplitude aberrations must be simulated using phase wrapping. This is compatible with operation at a single wavelength, but leads to chromatic effects with broadband light. Furthermore, the liquid crystals are designed to work with polarized light. In microscopes, SLMs are, therefore, best suited for use in laser illumination paths.

The purpose of adaptive elements is to compensate aberrations to ensure near-perfect imaging. In some situations, the effects of aberrations can instead be rectified through post-processing, such as through use of an aberrated PSF in deconvolution. However, in most microscopes, the presence of aberrations leads to decrease in signal-to-noise ratio and information loss, which cannot be recovered. For this reason, AO correction is usually the necessary course of action to obtain high-quality images.

## Theory of aberrations and image formation

In this section, we provide a brief summary of the main principles of aberration and imaging theory that support the following discussion.

In the context of AO, aberrations are usually expressed as the deviation of a wavefront from its ideal shape. This could be expressed in terms of differences in OPL, which depends upon the refractive index distribution along the path of a ray, or the phase aberration, which is the relative phase shift introduced across the wavefront by the variations in OPL within the optical medium. For a fixed OPL, the phase aberration Ψ would vary inversely with the wavelength of the light λ as

(1) $$\Psi =\frac{2\pi }{\lambda }\text{OPL}.$$

This spectral variation is an important consideration in fluorescence microscopes due to the Stokes shifts of fluorescent dyes and the possibility of multiple excitation and emission channels. Phase aberrations can be represented mathematically as the argument of a complex function defined in the pupil of the optical system. In general, this pupil function can be expressed in polar coordinates as

(2) P(r,θ)=A(r,θ)exp[iΨ(r,θ)],

where *i* is the imaginary unit, A(r,θ) represents the amplitude variations and Ψ(r,θ) is the phase aberration. For convenience, the circular pupil is usually defined to have unity radius, such that it exists for r≤1. As AO is mainly concerned with the correction of phase aberrations, it is common to neglect amplitude variations in analysis, although they may be significant in strongly aberrating specimens.

It is usual to represent the aberrations as a series of modes in the form

(3) $$\Psi (r,\theta )=\sum_{k}a_{k}X_{k}(r,\theta ),$$

where the modes $X_{k}(r,\theta )$ have coefficients $a_{k}$. These modes are often chosen for mathematical convenience to be an orthogonal set, such as the Zernike polynomials. These are particularly useful, as the different polynomials closely correspond to commonly observed aberrations, such as spherical, astigmatism and coma. Alternatively, the modes might be defined in terms of the modes produced by the adaptive element, or the aberrations that are predominant in a particular type of specimen. In all cases, the representation of aberrations in terms of a short series of modes simplifies considerably the design and control of an AO system.

Fluorescence microscopes are incoherent imaging systems, so the imaging process of the optical system alone can be modelled as a convolution of the specimen fluorescence distribution, denoted f(x), with the effective intensity PSF, $h_{\mathrm{eff}}(x)$, giving the image I(x) as

(4) $$I(x)=f\;(x)⊗h_{\mathrm{eff}}(x),$$

where ⊗ is the convolution operator, and x is the position vector. The effect of aberrations on the PSF of a conventional widefield fluorescence microscope can be represented (in the scalar approximation) as

(5) $$h_{\mathrm{eff}}(x)=|FT[P(r,\theta )]{|}^{2},$$

where FT is the Fourier transform. It is clear that the aberrations affect the form of the PSF and hence the quality of the image. In super-resolution microscopes, the effects are usually more complex, due to the more complicated imaging and processing steps.

## General consideration of aberrations in super-resolution microscopes

There has been extensive research on the implementation of AO in conventional resolution microscopes. This previous work provides useful background, but there are many ways in which super-resolution microscopes are affected differently by aberrations. The effects of aberrations on super-resolution microscopes can be considered in different ways:

- The microscope configuration on which the method is based: An understanding of how aberrations affect the corresponding conventional microscope provides a starting point for modelling aberration effects in the super-resolution microscope.
- Specific effects on the optical processes in the super-resolution system: These effects may be significantly different to those in conventional microscopes. For example, there may be greater sensitivity to particular aberration modes.
- Effects on data processing during or after image acquisition: Errors may occur if it is assumed that the image acquisition is diffraction limited when aberrations are present.

These effects will all be considered for SMS, STED and SIM imaging in the next sections.

## Single-molecule switching microscopy

The optical system of a standard SMS microscope is based around a conventional widefield fluorescence microscope. This consists of an illumination path and an imaging path. The illumination path is usually configured to provide uniform illumination over a region of the specimen, and expanded laser beams are regularly used for this purpose in order to provide the necessary illumination intensity at the correct wavelength. Certain SMS microscopes, such as STORM, use only one laser wavelength, although the other methods, such as PALM, require two or more wavelengths. In all of these cases, the microscope uses flood illumination, analogous to the lamp-based illumination of conventional fluorescence microscopes. It is well known that aberrations in the condenser path of a conventional microscope do not affect the final image quality, which is determined solely by the optics of the objective lens and imaging path. It follows that in a SMS microscope, aberration correction is only necessary in the imaging path. We note that light sheet illumination has also been used in some SMS microscopes, although the discussion of aberrations effects in these systems is outside the scope of this review [11,12].

The image in a SMS microscope is simply described by the convolution, as presented in Eq. (4) in the context of a conventional widefield microscope:

(6) $$I(x)=f\;(x)⊗h_{\mathrm{eff}}(x).$$

Each raw image acquired by the SMS microscope consists of a random selection of the fluorescence emitters in the specimen. As such, for each image, the function f(x) can be considered as a random collection of point objects and the overall image I(x) as a combination of randomly positioned PSFs. This observation provides useful prior knowledge for the design of an image-based AO scheme for SMS microscopes.

### Correction of aberrations in SMS microscopes

The fluorescence emission from the specimen is unpolarized, broadband and, if multiple labels are used, at a wide range of wavelengths. DMs are best suited for correction of this emission, due to their polarization and wavelength-independent operation. SMS microscopes have been implemented using DMs for predictive aberration correction [13] and feedback correction of specimen-induced aberrations [14]. The DM can also introduce a controlled amount of astigmatism to enable three-dimensional (3D) discrimination of emitter location [13,14].

SLMs are less well suited to correction in these microscopes, as they are usually configured to operate on one linear polarization state. However, some sophisticated methods have been developed that have potential for use in super-resolution microscopes [15]. The randomly polarized fluorophore emission can be separated into two orthogonal linear polarization states, each of which can be manipulated independently by different regions of the SLM [16]. This configuration provides versatility in the detection and interpretation of single emitter fluorescence.

### Effects of aberrations on image formation

There are four major steps in SMS microscopy that determine the nature of the final image representation:

- **Specimen preparation**: including the biological preparation and labelling protocols: The requirements for obtaining good SMS images can be considerably more stringent than those for conventional fluorescence imaging.
- **Data acquisition**: The properties of the acquired raw blinking images can be adjusted through control of the blink rate, which depends upon illumination conditions and chemical buffers. The fidelity of each emitter image is also affected by aberrations, which distort the PSF of the system.
- **Localization procedure**: Each emitter must be identified and fitted to a model PSF. The presence of aberrations in the imaging system will affect this step, if there is a mismatch between the system and model PSFs. There are two main consequences: either the fit may not meet the acceptance criteria and will be rejected or the fit is accepted, but with compromised localization precision or accuracy.
- **Image representation**: The localization procedure generates estimates of the emitter positions, along with the corresponding uncertainties. There are many different ways to represent these data in image format. As aberrations can affect the localization precision and accuracy, this might be taken into account when choosing a representation.

Aberrations have a major effect on two of these steps: the data acquisition and the localization procedure. The role of an AO system would be to remove aberrations before image acquisition, thus providing diffraction-limited emitter images and optimal localization.

### Implementations of adaptive optics

**Fig. 1.**
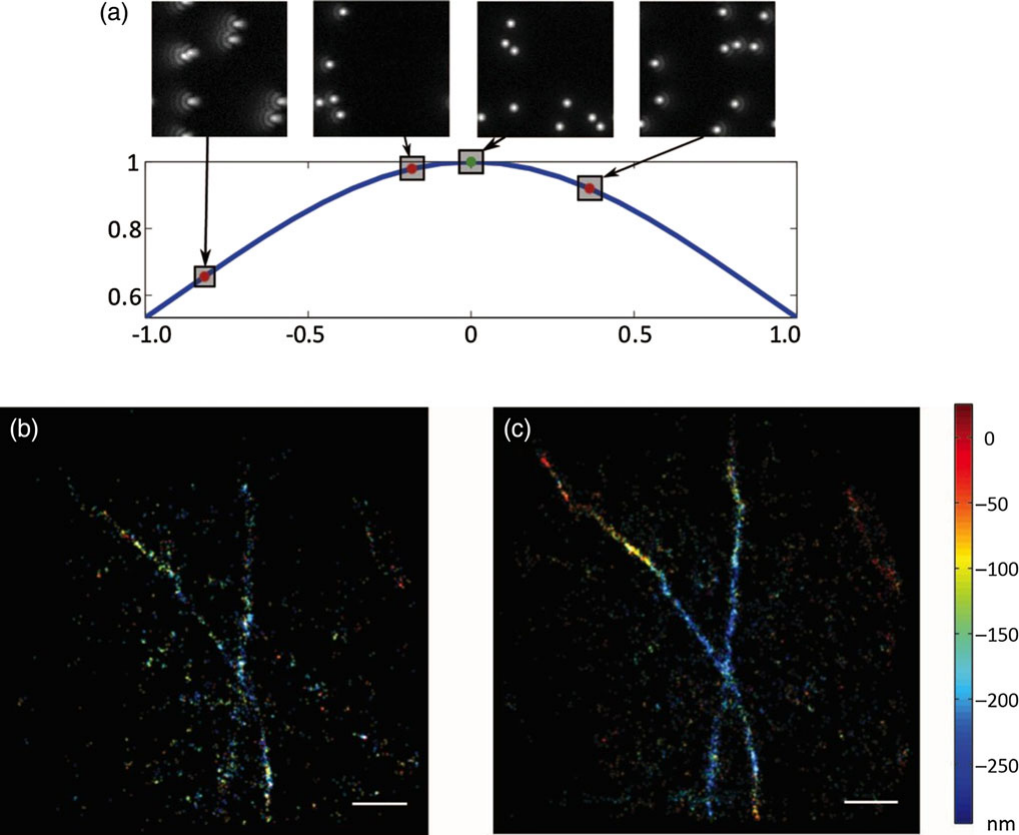
Aberration correction in a SMS microscope. (a) Principle of image-based optimization. For each mode used in the aberration correction procedure, different amplitudes are applied and their correspondent metric value is computed. Fitting of the resulting curve provides the coefficient that maximizes the metric and thus corrects the aberration mode. (b) 3D STORM image of Alexa 647-labelled microtubules obtained using the astigmatism method without aberration correction. (c) 3D STORM image of the same structure when aberrations were corrected; a significant increase in accepted fits is observed. In b and c, the images were taken 6 µm deep into the cell, and the *z* position is represented by the colour scale. (Images reproduced with permission from Ref. [14].) In b and c, scale bars are 1 µm.

Recent research has shown the benefit of aberration correction for SMS super-resolution microscopy. Burke *et al.* [14] implemented an AO system for feedback aberration correction in a STORM microscope. The fact that each individual blinking image is essentially a random collection of point objects enabled the design of an image-based correction system. These sensorless correction schemes have been demonstrated in various conventional resolution adaptive microscopes [3]. Aberrations were sequentially measured and corrected using a sequence of images at the start of the experiment (Fig. 1). The aberrations thus derived were then corrected using a DM, and the sequence of blinking images was acquired. Typically, the correction sequence could be completed in tens or hundreds of frames, whereas the STORM acquisition would require tens of thousands of frames. The correction procedure added only minor complexity to the imaging experiment.

Correction of aberrations when performing two-dimensional (2D) STORM a few micrometres into a cell led to an increase in the number of accepted fits and an improvement in the localization precision (Fig. 1). Considerable improvements between corrected and uncorrected images were seen in 3D STORM images, using an astigmatic imaging system to derive the *z* position of emitters. In this case, significant differences in the estimated *z* position were found, due to mismatch between the PSF of the system and the model PSF used in the localization procedure.

Other methods can also be employed to extract the *z* position of emitters in SMS microscopy. These include biplane imaging [17] and helical PSFs [18]; both of these methods, like astigmatic SMS microscopy, obtain *z* positions though a single objective lens. Other methods employ two opposing objectives in order to extract 3D information [19,20]. It is likely that aberrations affect each of these methods, although the various imaging mechanisms mean that the effects are likely to be different in each case. Further studies will reveal the relative robustness of each method.

If aberrations cannot be corrected (or if there are residual aberrations following adaptive compensation), then information about the aberrations could be incorporated into the localization procedure [21]. One option is to allow aberration mode coefficients to be free parameters to be estimated in the localization algorithm, although this would add considerably to computational complexity. Another option would be to measure the aberrations in some other way – perhaps using a wave front sensor – and then use this information in the PSF model for the fitting routine. In either case, the localization procedure would prove better than one where aberration effects are neglected.

## Stimulated emission depletion microscopy

The STED microscope consists essentially of three optical paths. The first is an illumination path, where a laser beam is focussed by the objective lens into the specimen to excite fluorescent markers. The second path is the detection path, whereby emitted fluorescence is collected by the objective lens and passed through to a pinhole photodetector. The third path consists of the depletion beam, which is shaped by a phase mask to provide the ring-shaped beam with zero intensity at the centre. The illumination and detection paths effectively form together a confocal scanning microscope. The optics of these paths have an effect on the image quality of the STED microscope, but the resolution and efficiency are determined primarily by the depletion beam.

There are two main common implementations of STED microscopy. The most widely used provides 2D resolution enhancement through the use of a helicoidal phase mask in the depletion beam. This mask creates a phase vortex that ensures zero intensity along the optical axis. The other common implementation provides 3D resolution enhancement by creating a depletion focus where a point of zero intensity is surrounded in all directions by light. Both of these configurations are affected by aberrations, although 3D STED is more sensitive and therefore more difficult to implement in practical situations. In some situations, both the 2D and 3D patterns are combined by superimposing two beams with opposite circular polarizations [9,22].

Image formation in the STED microscope is expressed by the convolution

(7) $$I(x)=f\;(x)⊗h_{\mathrm{eff}}(x).$$

The STED microscope operates in many ways like a scanning confocal microscope. Like the confocal fluorescence microscope, the effective PSF is described by the product of the excitation PSF and the detection PSF [23]:

(8) $$h_{\mathrm{eff}}(x)=h_{\mathrm{exc}}(x)h_{\det}(x).$$

The depletion beam has the effect of restricting the size of $h_{\mathrm{exc}}(x)$ by suppressing the fluorescence. We can, therefore, use a suppression factor [24] given by

(9) $$\eta (x)=\exp\;\left[\frac{-\ln(2)I_{\mathrm{dep}}(x)}{I_{S}}\right],$$

where $I_{\mathrm{dep}}(x)$ is the depletion beam intensity, and $I_{S}$ is the value of the intensity that reduces the fluorescence emission to a half of its original value. The STED PSF can then be expressed in terms of the confocal PSF $h_{\mathrm{conf}}(x)$ of the equivalent microscope as

(10) $$h_{\mathrm{eff}}(x)=\eta (x)h_{\mathrm{conf}}(x).$$

The exponential nature of the suppression factor means that the size of the effective PSF, and hence the resolution, is predominantly determined by $I_{\mathrm{dep}}(x)$. Understanding the effects of aberrations on the depletion spot is, therefore, of the greatest importance in determining the imaging properties of the STED microscope.

### Effects of aberrations on the depletion beam

**Fig. 2.**
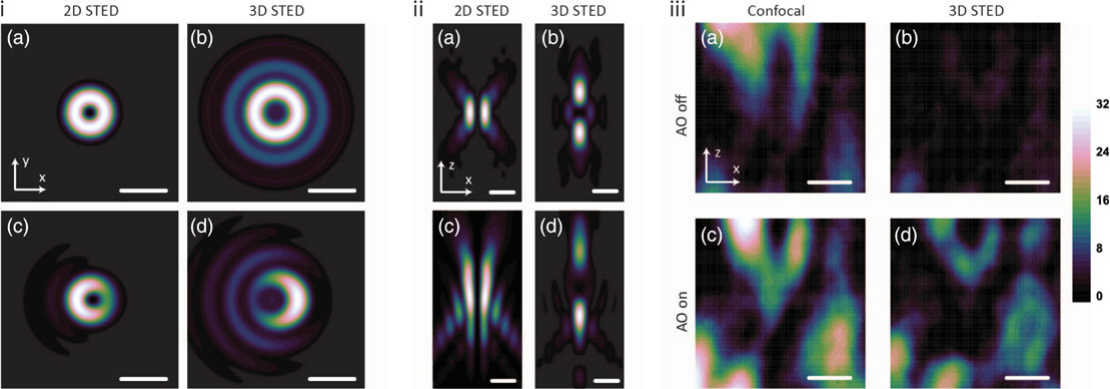
Effect of aberrations on STED microscopy. (i) Calculations of the effect of coma in the focal plane of STED depletion profiles. (a and b) Ideal focal plane intensity distributions of STED beams. (c and d) Focal plane intensity distributions of STED beams, where coma affects the shape of the PSF. (ii) Calculations of the effect of primary spherical aberration in the *xz* plane of STED depletion profiles. (a and b) Ideal focal plane intensity distributions of STED beams. (c and d) Focal plane intensity distributions of STED beams, where primary spherical aberration affects the shape of the PSF. (iii) Experimental demonstration of AO for STED microscopy. (a and b) Respectively, confocal and 3D STED images of Atto647N-labelled synaptic boutons of dopaminergic neurons located 15 µm into a *Drosophila* brain, without aberration correction. (c and d) Respectively, confocal and 3D STED images of the same structures as in (iii) a and b with aberration correction. In all images, scale bars are 1 µm.

The most important property of the depletion beam is the contrast between the point of minimum intensity (which ideally should be zero) and the surrounding ring of high-intensity illumination. The generation of such a beam requires a careful choice of phase mask and polarization properties; errors in either cause an increase in the value of the intensity minimum. The depletion beam is also susceptible to phase aberrations, where the specific effects depend upon the combination of the STED configuration and the aberration modes that are present [25,26] (Fig. 2). Considering their effects on STED microscopes, aberrations can be classified into two categories:

- Modes that distort the focal distribution but maintain the zero intensity. These modes have some effect on the resolution through distortion of the depletion beam, but the microscope could still show STED resolution enhancement. For large aberrations, the ring can open up, which means that no significant STED resolution enhancement can occur. In this case, the image would appear much like of a confocal microscope.
- Modes that fill in the zero intensity. By increasing the illumination intensity in the centre of the ring, excited fluorophores there are also depleted, leading to a reduction in the measured fluorescence. In this case, there would be a significant loss of image resolution and signal level. The resolution would be similar to a confocal microscope, but with very low efficiency.

Both categories of aberrations are detrimental to the performance of the STED microscope, although the second set is in many ways more critical. A small increase in the intensity minimum would in most cases negate any benefit of resolution enhancement, as the fluorescence emission would drop significantly. The corresponding fall in signal-to-noise ratio would render images unusable. It is, therefore, essential that zero intensity be maintained in the depletion profile for effective STED imaging.

### Effects of aberrations on the illumination and detection paths

The illumination and detection paths of a STED microscope form a confocal scanning microscope, and the effects of aberrations on these paths alone are identical to those experienced by a confocal fluorescence microscope [27]. Whilst this aspect is less critical, there are still effects on the performance of the STED microscope. In particular, the excitation focus is enlarged in the presence of aberrations, leading to a greater proportion of fluorescence excitation outside of the focal plane. This can lead to increased background fluorescence, as the out of focus fluorescence will not be suppressed efficiently by the depletion focus. Aberrations in the detection path mean that fluorescence collection by the confocal pinhole is reduced, and the overall efficiency of the microscope decreases.

### Correction of aberrations in STED microscopy

As all three beam paths suffer the same specimen-induced aberrations, it is desirable to use a single adaptive element that would be placed in the common beam path. As the wavelengths of the excitation, fluorescence and depletion beams are all different (and as multiple laser and fluorophore emission might be employed), a DM is best suited for this purpose. It is also useful to use a SLM in the depletion beam path, as it can provide both the phase mask for shaping the focus and fine aberration correction for the more sensitive depletion beam [28,29]. It is possible to correct the excitation beam with a second SLM, as the SLM will function well with the laser illumination [29], although this would not provide full correction of all paths.

Aside from distortions of the different laser foci, it is also important that the foci are precisely co-aligned to ensure that the zero intensity point of the depletion beam overlaps with the centre of the excitation focus in three dimensions. This procedure is aided by the use of a SLM, as automated feedback routines can be implemented using image optimization [29,30]. The SLM also permits easy modification of the phase mask properties, such as diameter and phase magnitude, which provides considerable versatility when compared with conventional fixed phase plates. Positioning of the phase mask is also important, as errors in the position could be misinterpreted as coma aberrations [31,32]. These adaptive feedback procedures simplify considerably the routine alignment of the microscope.

Correction of aberrations has been implemented in adaptive STED microscopes using image-based sensorless correction [29]. Correction was implemented through the optimization of an image quality metric that combined image brightness and sharpness. This enabled the recovery of STED performance that had been compromised, both in terms of resolution and signal level, by specimen-induced aberrations at focussing depths up to 25 μm. Further developments of aberration measurement and correction methods will extend the operating depth of adaptive STED microscopes (Fig. 2iii).

## Super-resolution structured illumination microscopy

The imaging path in SIM is similar to a widefield fluorescence microscope. The widefield microscope usually includes a Köhler illumination system, which provides flood illumination over the field of interest. In SIM, this is replaced by a system that projects a sinusoidal illumination pattern, which should be reproduced with high fidelity at the focal plane, within the specimen. This pattern is rotated and translated to a number of different positions in order to acquire a sequence of images that is processed to derive the super-resolution image [33].

The fluorescence emission distribution is given by the product of the illumination pattern, $I_{\mathrm{ill}}(x)$, and the fluorescence distribution, f(x), so that the acquired image is

(11) $$I(x)=[I_{\mathrm{ill}}(x)f\;(x)]⊗h_{\mathrm{eff}}(x).$$

As in the widefield microscope, aberrations affect the image formation via distortion of the imaging PSF $h_{\mathrm{eff}}(x)$. Another important effect in SIM concerns the reproduction of the illumination pattern, which is produced through the same optics as the imaging path and is therefore also affected by aberrations. For optimum performance, coherent illumination conditions are chosen (or partially coherent conditions that are nearly fully coherent, for practical reasons). In the fully coherent approximation, which we use for the sake of illustration, the illumination pattern is given by

(12) $$I_{\mathrm{ill}}(x)={\left|\frac{1}{2}[1+\cos(x\cdot p+\phi )]⊗h_{\mathrm{amp}}(x)\right|}^{2},$$

where p and ϕ define the orientation and position of the sinusoidal illumination pattern, respectively. $h_{\mathrm{amp}}(x)$ is the amplitude (rather than intensity) PSF [34], defined as

(13) $$h_{\mathrm{amp}}(x)=\mathrm{FT}[P(r)],$$

where P is the pupil function, and r is the coordinate vector in the pupil plane. Application of the Fourier convolution theorem to Eq. (12) gives

(14) $$I_{\mathrm{ill}}(x)={\left|F\;T^{-1}\left\{\frac{1}{2}\left[\delta (r)+\frac{e^{-i\phi }}{2}\delta (r-p)+\frac{e^{i\phi }}{2}\delta (r+p)\right]P(r)\right\}\right|}^{2},$$

where p is chosen to have magnitude of 1 so that the delta functions are positioned in the centre and at the edge of the pupil. It is clear from this expression that illumination is present only at three points in the pupil. This has important consequences when considering the effects of aberrations on the reproduction of the sinusoidal illumination pattern.

### Effects of aberrations on the structured illumination

**Fig. 3.**
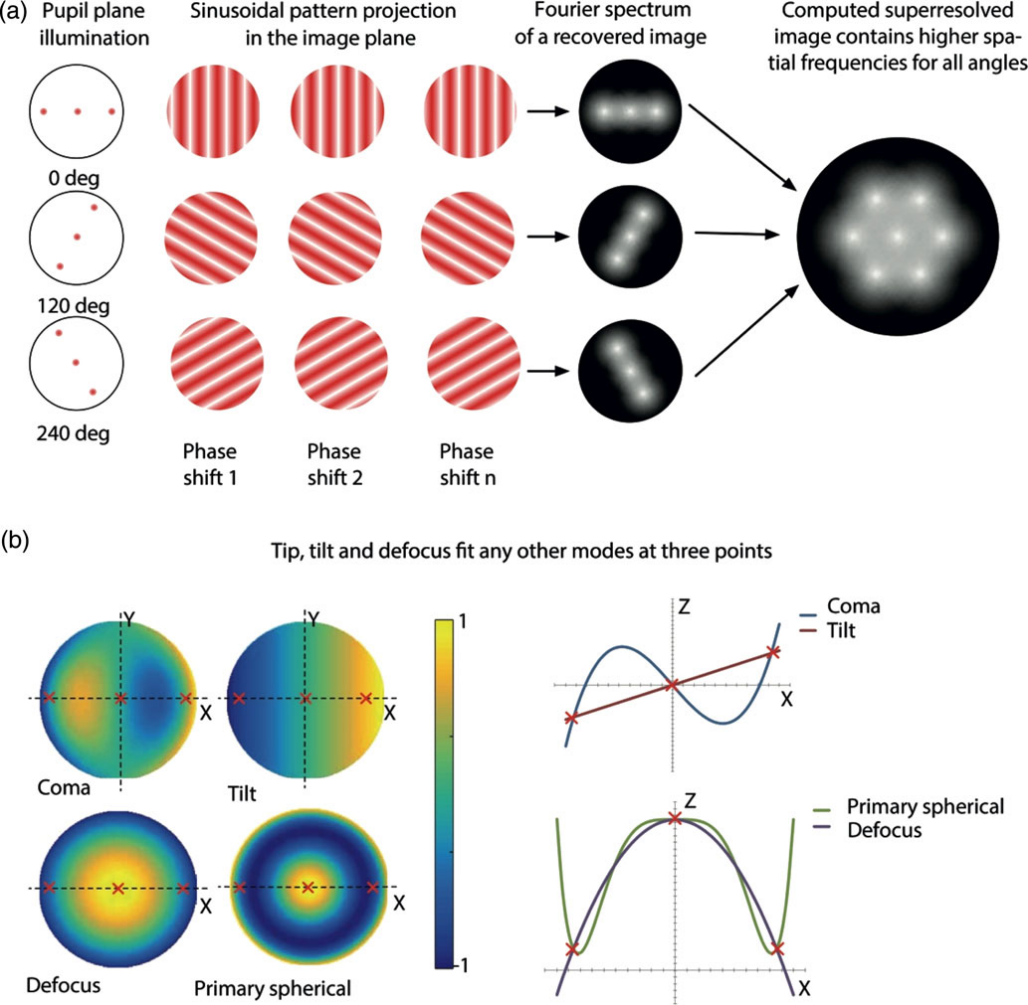
Effect of aberrations on SIM. (a) Schematic of the principles of SIM. (b) Schematic showing how aberrations in the illumination pattern are equivalent to an offset in the spatial coordinates of the intensity focal distribution. In these examples, coma creates a lateral shift equivalent to tilt; spherical aberration shifts along the axis in the same way as defocus.

The spatial frequency of the sinusoidal pattern is chosen to be close to the cut-off frequency of the optical system, in order to achieve the maximum resolution in the reconstructed image. As seen in Eq. (14), generation of a single instance of this pattern requires the illumination of three points in the pupil of the objective lens: one at the centre and two diametrically opposed at the edge. For each raw image, it follows that only the phase values at these three points have an effect on the reproduction of the pattern in the specimen. The lateral position of the pattern depends upon the relative phase between these three points (Fig. 3). It is straightforward to understand this by considering the action of a tilting mirror in the pupil, which would add a phase offset proportional to the distance across the mirror.

The pattern can also be defocussed if the phase of the two peripheral points is adjusted in tandem relative to the central point (Fig. 3). This can be understood by considering the application of a rotationally symmetric defocus phase function across the pupil.

In the typical sequence of images required for super-resolution SIM, the pattern is rotated to at least three different orientations. This leads to a collection of points around the periphery of the pupil, where illumination is required, in addition to the point in the middle. This means that the phase at all points of the pupil that are not either at one of the edge points or at the middle has no effect on illumination pattern. The illumination patterns should, therefore, be immune to some aberration modes that have little phase variation around the edge of the pupil. Furthermore, the rotationally symmetric nature of spherical aberration means that it has the main effect of refocussing the pattern (Fig. 3). Other modes may simply affect the spatial position of the patterns.

### Effects of aberration in imaging

Aberrations affect the imaging path, just as they would do in a widefield microscope. As expressed in Eq. (11), the object is in effect formed by the illumination pattern multiplied by specimen fluorescence distribution. The image formed on the camera is the convolution of this with the imaging PSF, which is distorted by aberrations, and hence leads to a blurred image. Previous work on AO for sectioning (rather than super-resolution), SIM has shown that the imaging properties of such a system are dominated by its ability to reliably image the high spatial frequency of the pattern, even though other frequencies are also present [35]. This is expected also to be the case in super-resolution SIM. It was also seen that a certain class of aberrations modes could be defined that had no effect on the pattern and little effect on the processed images.

### Correction of aberrations in SIM

The performance of SIM relies upon the fidelity of both illumination and detection paths. The correction in these paths could be performed simultaneously using a single DM, which would be ideal for the fluorescence emission [36]. Other methods could, however, be used in the illumination path. SLMs are possible candidates for generating the illumination patterns, whilst providing adaptability to deal with any aberrations. As only seven points of illumination are actually needed in the illumination pupil, another option is to use seven appropriately positioned optical fibres [37]. Aberration correction could then be implemented by adjusting the relative phase from each fibre. In the specific case of spherical aberration, the rotational symmetry means that only the phase of the centrally positioned fibre needs be modulated.

A level of correction could be performed in post-processing [36], particularly if the aberrations predominantly affect the lateral shifts of the illumination patterns. The errors in the shifts could be measured by observing the wavefront aberrations during imaging or estimated thereafter, permitting compensation in the derivation of the super-resolved images. Furthermore, known aberrations could be incorporated into the reconstruction process to improve the final image quality.

## Concluding remarks

The correction of aberrations through AO is becoming widespread in high-resolution microscopy. However, the extension of AO to super-resolution microscopy presents further challenges, due to the higher sensitivity to aberrations exhibited by these techniques. Each of the methods covered by this review – SMS, STED and SI microscopy – is affected by aberrations in different ways, and understanding these effects is an important step towards designing appropriate AO correction systems. The potential for use of AO in these systems is clear, and initial developments have shown the benefits that can be obtained when imaging deeper in specimens. Furthermore, all the super-resolution techniques that share the conceptual framework of reversible saturable optical fluorescence transitions [38] microscopy would benefit from AO in a similar fashion as STED, as the technical aspects of their optical set-ups are similar.

In addition to the general improvement in imaging quality parameters, such as signal-to-noise ratio and spatial resolution, AO may also facilitate the application of super-resolution methods to living specimens, where phototoxic effects may compromise the biological relevance of the image. By correcting aberrations, the peak laser intensity used in the experiment is expected to be lower for a given target resolution and signal-to-noise ratio, increasing the biochemical and morphological stability of the specimen. Further developments in aberration measurement and correction tailored to these methods will support a new wave of applications for super-resolution imaging in thick specimens.

## Funding

The authors acknowledge support from the Wellcome Trust (095927/A/11/Z) and Medical Research Council (MR/K01577X/1). B.P. is supported by the Royal Society, through a University Research Fellowship. Funding to pay the Open Access publication charges for this article was provided by the Wellcome Trust.

## References

[1] Booth M J (2007) Adaptive optics in microscopy. Philos. Trans. A Math. Phys. Eng. Sci. 365: 2829–2843. 17855218 10.1098/rsta.2007.0013

[2] Kubby J A (2013) Adaptive Optics for Biological Imaging (CRC Press, Boca Raton, FL).

[3] Booth M J (2014) Adaptive optical microscopy: the ongoing quest for a perfect image. Light Sci. Appl. 3: e165.

[4] Betzig E, Patterson G H, Sougrat R, Lindwasser O W, Olenych S, Bonifacino J S, Davidson M W, Lippincott-Schwartz J, Hess H F (2006) Imaging intracellular fluorescent proteins at nanometer resolution. Science 313: 1642–1645. 16902090 10.1126/science.1127344

[5] Hess S T, Girirajan T P, Mason M D (2006) Ultra-high resolution imaging by fluorescence photoactivation localization microscopy. Biophys. J. 91: 4258–4272. 16980368 10.1529/biophysj.106.091116 PMC1635685

[6] Rust M J, Bates M, Zhuang X (2006) Sub-diffraction-limit imaging by stochastic optical reconstruction microscopy (STORM). Nat. Methods 3: 793–795. 16896339 10.1038/nmeth929 PMC2700296

[7] Hell S W, Wichmann J (1994) Breaking the diffraction resolution limit by stimulated emission: stimulated-emission-depletion fluorescence microscopy. Opt. Lett. 19: 780–782. 19844443 10.1364/ol.19.000780

[8] Gustafsson M G (2000) Surpassing the lateral resolution limit by a factor of two using structured illumination microscopy. J. Microsc. 198: 82–87. 10810003 10.1046/j.1365-2818.2000.00710.x

[9] Wildanger D, Medda R, Kastrup L, Hell S (2009) A compact STED microscope providing 3D nanoscale resolution. J. Microsc. 236: 35–43. 19772534 10.1111/j.1365-2818.2009.03188.x

[10] Gottfert F, Wurm C A, Mueller V, Berning S, Cordes V C, Honigmann A, Hell S W (2013) Coaligned dual-channel STED nanoscopy and molecular diffusion analysis at 20 nm resolution. Biophys. J. 105: L01–L03. 23823248 10.1016/j.bpj.2013.05.029 PMC3699760

[11] Hu Y, Zhu Q, Elkins K, Tse K, Li Y, Fitzpatrick J, Verma I, Cang H (2013) Light-sheet Bayesian microscopy enables deep-cell super-resolution imaging of heterochromatin in live human embryonic stem cells. Opt. Nanoscopy 2: 7. 10.1186/2192-2853-2-7 PMC5082751 27795878

[12] Hu Y S, Zimmerley M, Li Y, Watters R, Cang H (2014) Single-molecule super-resolution light-sheet microscopy. ChemPhysChem 15: 577–586. 24615819 10.1002/cphc.201300732

[13] Izeddin I, El Beheiry M, Andilla J, Ciepielewski D, Darzacq X, Dahan M (2012) PSF shaping using adaptive optics for three-dimensional single-molecule super-resolution imaging and tracking. Opt. Express 20: 4957–4967. 22418300 10.1364/OE.20.004957

[14] Burke D, Patton B, Huang F, Bewersdorf J, Booth M J (2015) Adaptive optics correction of specimen-induced aberrations in single-molecule switching microscopy. Optica 2: 177–185.

[15] Jesacher A, Ritsch-Marte M, Piestun R (2015) Three-dimensional information from two-dimensional scans: a scanning microscope with postacquisition refocusing capability. Optica 2: 210–213.

[16] Roider C, Jesacher A, Bernet S, Ritsch-Marte M (2014) Axial super-localisation using rotating point spread functions shaped by polarisation-dependent phase modulation. Opt. Express 22: 4029–4037. 24663724 10.1364/OE.22.004029

[17] Juette M F, Gould T J, Lessard M D, Mlodzianoski M J, Nagpure B S, Bennett B T, Hess S T, Bewersdorf J (2008) Three-dimensional sub-100 nm resolution fluorescence microscopy of thick samples. Nat. Methods 5: 527–529. 18469823 10.1038/nmeth.1211

[18] Pavani S R P, Thompson M A, Biteen J S, Lord S J, Liu N, Twieg R J, Piestun R, Moerner W E (2009) Three-dimensional, single-molecule fluorescence imaging beyond the diffraction limit by using a double-helix point spread function. Proc. Natl. Acad. Sci. USA 106: 2995–2999. 19211795 10.1073/pnas.0900245106 PMC2651341

[19] Shtengel G, Galbraith J A, Galbraith C G, Lippincott-Schwartz J, Gillette J M, Manley S, Sougrat R, Waterman C M, Kanchanawong P, Davidson M W, Fetter R D, Hess H F (2009) Interferometric fluorescent super-resolution microscopy resolves 3D cellular ultrastructure. Proc. Natl. Acad. Sci. USA 106: 3125–3130. 19202073 10.1073/pnas.0813131106 PMC2637278

[20] Aquino D, Schonle A, Geisler C, Middendorff C V, Wurm C A, Okamura Y, Lang T, Hell S W, Egner A (2011) Two-color nanoscopy of three-dimensional volumes by 4Pi detection of stochastically switched fluorophores. Nat. Methods 8: 353–359. 21399636 10.1038/nmeth.1583

[21] Liu S, Kromann E B, Krueger W D, Bewersdorf J, Lidke K A (2013) Three dimensional single molecule localization using a phase retrieved pupilfunction. Opt. Express 21: 29462–29487. 24514501 10.1364/OE.21.029462 PMC3867195

[22] Lenz M O, Sinclair H G, Savell A, Clegg J H, Brown A C, Davis D M, Dunsby C, Neil M A, French P M (2014) 3-D stimulated emission depletion microscopy with programmable aberration correction. J. Biophotonics 7: 29–36. 23788459 10.1002/jbio.201300041

[23] Gu M (2000) Advanced Optical Imaging Theory (Springer-Verlag, Berlin Heidelberg).

[24] Harke B, Keller J, Ullal C K, Westphal V, Schönle A, Hell S W (2008) Resolution scaling in STED microscopy. Opt. Express 16: 4154–4162. 18542512 10.1364/oe.16.004154

[25] Deng S, Liu L, Cheng Y, Li R, Xu Z (2010) Effects of primary aberrations on the fluorescence depletion patterns of STED microscopy. Opt. Express 18: 1657–1666. 20173993 10.1364/OE.18.001657

[26] Deng S, Liu L, Cheng Y, Li R, Xu Z (2009) Investigation of the influence of the aberration induced by a plane interface on STED microscopy. Opt. Express 17: 1714–1725. 19189001 10.1364/oe.17.001714

[27] Booth M J, Neil M A A, Juškaitis R, Wilson T (2002) Adaptive aberration correction in a confocal microscope. Proc. Natl. Acad. Sci. USA 99: 5788–5792. 11959908 10.1073/pnas.082544799 PMC122854

[28] Auksorius E, Boruah B R, Dunsby C, Lanigan P M P, Kennedy G, Neil M A A, French P M W (2008) Stimulated emission depletion microscopy with a supercontinuum source and fluorescence lifetime imaging. Opt. Lett. 33: 113–115. 18197209 10.1364/ol.33.000113

[29] Gould T J, Burke D, Bewersdorf J, Booth M J (2012) Adaptive optics enables 3D STED microscopy in aberrating specimens. Opt. Express 20: 20998–21009. 23037223 10.1364/OE.20.020998 PMC3635694

[30] Gould T J, Kromann E B, Burke D, Booth M J, Bewersdorf J (2013) Auto-aligning stimulated emission depletion microscope using adaptive optics. Opt. Lett. 38: 1860–1862. 23722769 10.1364/OL.38.001860 PMC3749882

[31] Patton B R, Burke D, Vrees R, Booth M J (2015) Is phase-mask alignment aberrating your STED microscope? Methods Appl. Fluoresc. 3: 024002. 10.1088/2050-6120/3/2/024002 29148484

[32] Kromann E B, Gould T J, Juette M F, Wilhjelm J E, Bewersdorf J (2012) Quantitative pupil analysis in stimulated emission depletion microscopy using phase retrieval. Opt. Lett. 37: 1805–1807. 22660035 10.1364/OL.37.001805 PMC3558916

[33] Gustafsson M G, Shao L, Carlton P M, Wang C J, Golubovskaya I N, Cande W Z, Agard D A, Sedat J W (2008) Three-dimensional resolution doubling in wide-field fluorescence microscopy by structured illumination. Biophys. J. 94: 4957–4970. 18326650 10.1529/biophysj.107.120345 PMC2397368

[34] Wilson T, Sheppard C (1984) Theory and Practice of Scanning Optical Microscopy (Academic Press, Inc., London, New York).

[35] Débarre D, Botcherby E J, Booth M J, Wilson T (2008) Adaptive optics for structured illumination microscopy. Opt. Express 16: 9290–9305. 18575493 10.1364/oe.16.009290

[36] Thomas B, Wolstenholme A, Chaudhari S N, Kipreos E T, Kner P (2015) Enhanced resolution through thick tissue with structured illumination and adaptive optics. J. Biomed. Opt. 20: 026006-026006. 10.1117/1.JBO.20.2.026006 25714992

[37] Arigovindan M, Sedat J W, Agard D A (2012) Effect of depth dependent spherical aberrations in 3D structured illumination microscopy. Opt. Express 20: 6527–6541. 22418536 10.1364/OE.20.006527

[38] Grotjohann T, Testa I, Leutenegger M, Bock H, Urban N T, Lavoie-Cardinal F, Willig K I, Eggeling C, Jakobs S, Hell S W (2011) Diffraction-unlimited all-optical imaging and writing with a photochromic GFP. Nature 478: 204–208. 21909116 10.1038/nature10497

